# Poly[[tetra­aqua­(μ_3_-4-hy­droxy­pyridine-2,6-di­carboxyl­ato)di-μ_2_-oxalato-dipraseodymium(III)] 4.29-hydrate]

**DOI:** 10.1107/S2414314625004560

**Published:** 2025-05-30

**Authors:** Aaqib Khurshid, Apinpus Rujiwatra, Thammanoon Chuasaard

**Affiliations:** a Department of Chemistry Faculty of Science Chiang Mai University,Chiang Mai 50200 Thailand; b Materials Science Research Center Faculty of Science Chiang Mai University,Chiang Mai 50200 Thailand; c Office of Research Administration Chiang Mai University,Chiang Mai 50200 Thailand; Purdue University, USA

**Keywords:** praseodymium, chelidamic acid, coordination polymer, hydrogen bonding, crystal structure

## Abstract

The coordination polymer of crystal formula [Pr^III^_2_(C_7_H_2_NO_5_)(C_2_O_4_)_2_(H_2_O)_4_]·4.29H_2_O or [Pr^III^_2_(HCAM)(ox)_2_(H_2_O)_4_]·4.29H_2_O was synthesized from praseodymium(III) nitrate in water using chelidamic acid (H_3_CAM) and oxalic acid (H_2_ox). There are two Pr^III^ atoms in the asymmetric unit. One metal ion has nine-fold coordination from one pyridyl nitro­gen and eight oxygen atoms from one HCAM^2−^, two ox^2−^, and two coordinating water mol­ecules leading to the formation of a tricapped trigonal {Pr^III^NO_8_} prism. The other metal ion is coordinated by ten oxygen atoms from two HCAM^2−^, two ox^2−^, and two coordinating water mol­ecules, forming a bicapped square {Pr^III^O_10_} unit. The HCAM^2−^ linker has a μ_2_-κ^2^:κ^1^ chelating coordination mode *via* its carboxyl­ates and N-pyridyl donors, linking the {Pr^III^NO_8_} and {Pr^III^O_10_} units into infinite chains. The μ_2_-κ^1^:κ^1^:κ^1^:κ^1^ bridging carboxyl­ates of the ox^2−^ linkers connect adjacent chains into sheets in the *ac* plane. Adjacent layers further aggregate through inter­molecular hydrogen bonding, most of which involves the water mol­ecules of crystallization, and π–π inter­actions to form a tri-periodic supra­molecular framework. Some of the co-crystallizing water mol­ecules as well as one of the metal-coordinating water mol­ecules are disordered.

## Abstract 2

The title compound [Pr^III^_2_(HCAM)(ox)_2_(H_2_O)_4_]·4.29H_2_O features a two-dimensional coordination polymeric framework of Pr^III^ with partially deprotonated chelidamic acid (HCAM^2−^) and oxalate (ox^2−^) linkers. The two-dimensional sheets further assemble through hydrogen bonding and π–π inter­actions into three-dimensional supra­molecular architecture.

## Graphical abstract

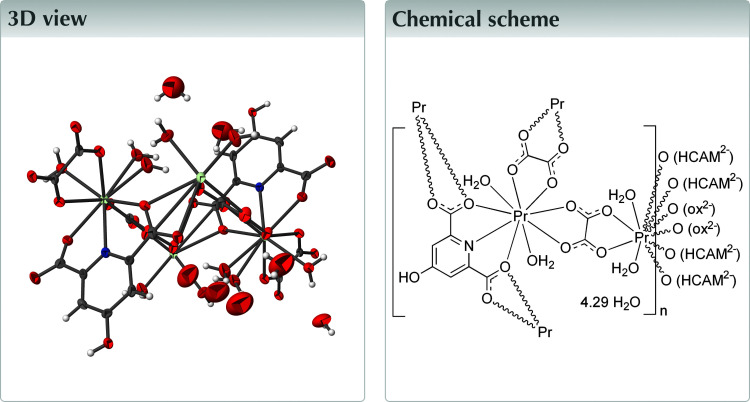

## Structure description

A coordination polymer (CP) is a metal coordination compound with repeating coordination entities consisting of metal ions or clusters - the nodes - that are connected through coordinating ligands into an infinite solid-state assembly with different periodcities. Lanthanide–CPs (*Ln*^III^–CPs) in which the metal nodes are lanthanide ions are of inter­est as they combine the characteristics of CPs such as an often high chemical and thermal stability and a capability to be tailor-made to include functional groups with the lanthanide’s unique properties based on their various coordination geometries and characteristic optical and magnetic properties (Li *et al.*, 2015 ▸; Bünzli, 2014 ▸). These merits can provide *Ln^I^*^II^–CPs with fascinating structures and functions. Their well-known applications include catalysis (Zhang *et al.*, 2021 ▸), luminescent sensing (Wang *et al.*, 2023 ▸) and gas adsorption (Roy *et al.*, 2014 ▸), to name just a few. All *Ln*^III^ ions have a high oxophilicity, so organic polycarboxyl­ates are commonly employed as organic linkers in *Ln*^III^–CPs (Bünzli, 2014 ▸). The high coordination numbers and flexible coordination geometries of *Ln^I^*^II^, as well as a lack of directionality of *Ln*—O bonds do, however, make it difficult to predict the exact nature of the resultant polymeric framework, which is also greatly affected by the choice of solvent used during synthesis, which is often incorporated into the coordination polymer structure (Bünzli, 2014 ▸; Patra & Pal, 2025 ▸). Owing to their often labile nature, the solvent mol­ecules tend to exhibit disorder, even though they can consolidate the framework structure by promoting inter­molecular hydrogen-bonding inter­actions. The introduction of hydrogen-bond-promoting groups such as hydroxyl on an organic linker is therefore expected to support the framework structure of CPs by transfixing the solvent mol­ecules. Chelidamic acid (H_3_CAM) containing an –OH group on a pyridyl ring was selected to be the organic linker to bind with praseodymium(III) (Pr^III^) in this work. Oxalic acid (H_2_ox) was also used as another small organic linker to help prevent the coordination of labile solvent mol­ecules to the coordination sphere of Pr^III^ and to provide the possibility of obtaining a new Pr^III^–CP structure with high dimensionality.

**Figure 1:**
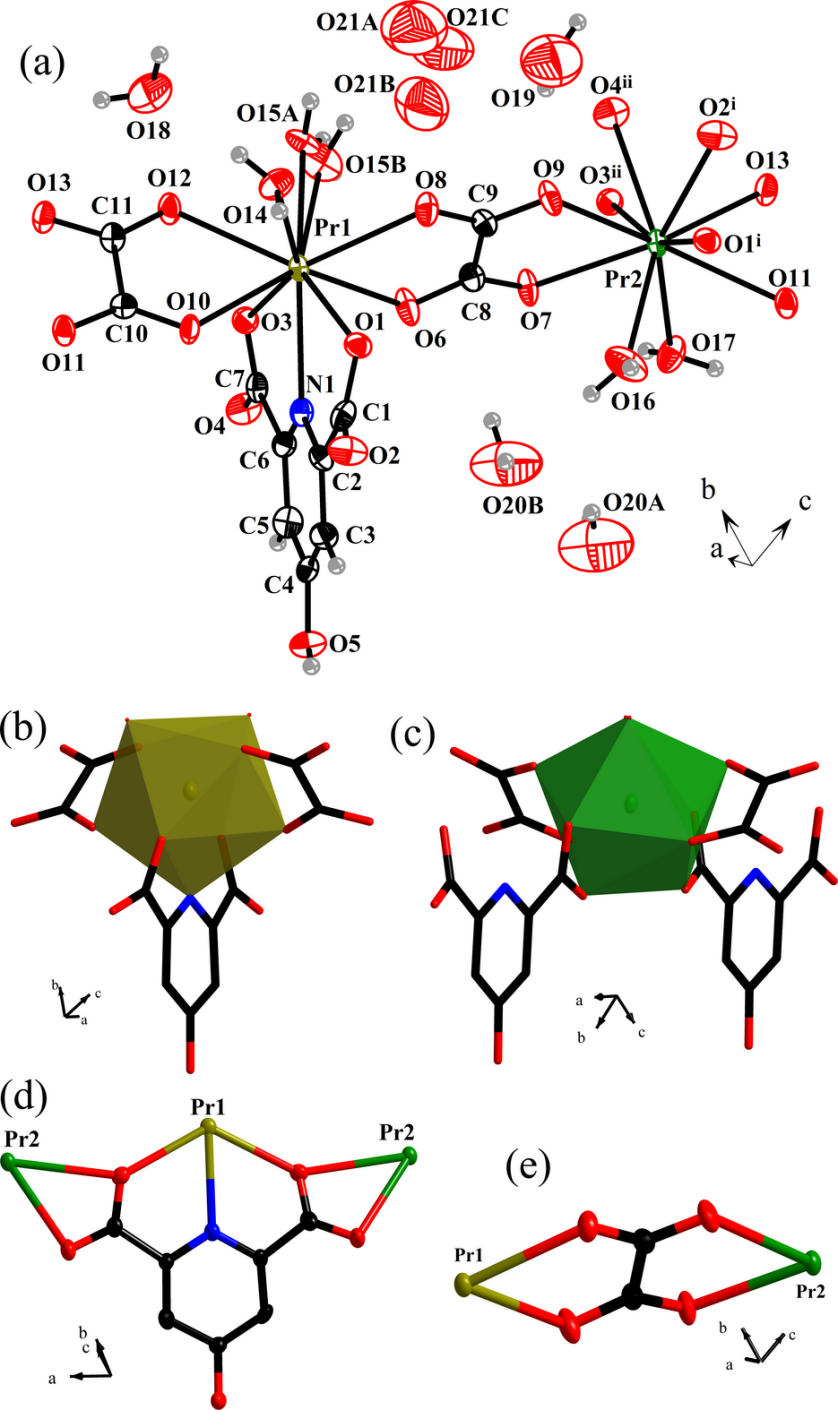
A depiction of (*a*) an extended asymmetric unit of title compound drawn using 50% probability ellipsoids, (*b*) *TPRS*-{Pr^III^NO_8_} building unit of Pr1, (*c*) *SAPRS*-{Pr^III^O_10_} building unit of Pr2, (*d*) coordination mode adopted by HCAM^2−^, and (*e*) coordination mode adopted by ox^2−^. [Symmetry codes: (i) −*x*, −*y* + 1, −*z* + 1; (ii) −*x* + 1, −*y* + 1, −*z* + 1].

The asymmetric unit of the title compound, *i.e.* [Pr^III^_2_(HCAM)(ox)_2_(H_2_O)_4_]·4.29H_2_O, is made up of two Pr^III^ ions (Pr1 and Pr2), one complete HCAM^2−^ dianion, two ox^2−^ dianions, four metal-coordinating water mol­ecules (two at each Pr^III^ ion), and approximately four and a third co-crystallizing water mol­ecules (Fig. 1 ▸*a*). One of the coordinating water mol­ecules is disordered over two sites (O15*A* and O15*B*). Two of the four crystallizing water mol­ecule are not disordered (O18 and O19), whilst O20 is hydrogen bonded to its own symmetry equivalent by inversion, inducing site disorder splitting to O20*A* and O20*B*, each of which exhibit 50% occupancy. The other co-crystallizing water mol­ecules are extensively disordered and were refined over three partially occupied sites (O21*A*, O21*B* and O21*C*), which share a total site occupancy of 1.294 water mol­ecules. The correlated disorder prevents their hydrogen atoms to be resolved.

**Figure 2:**
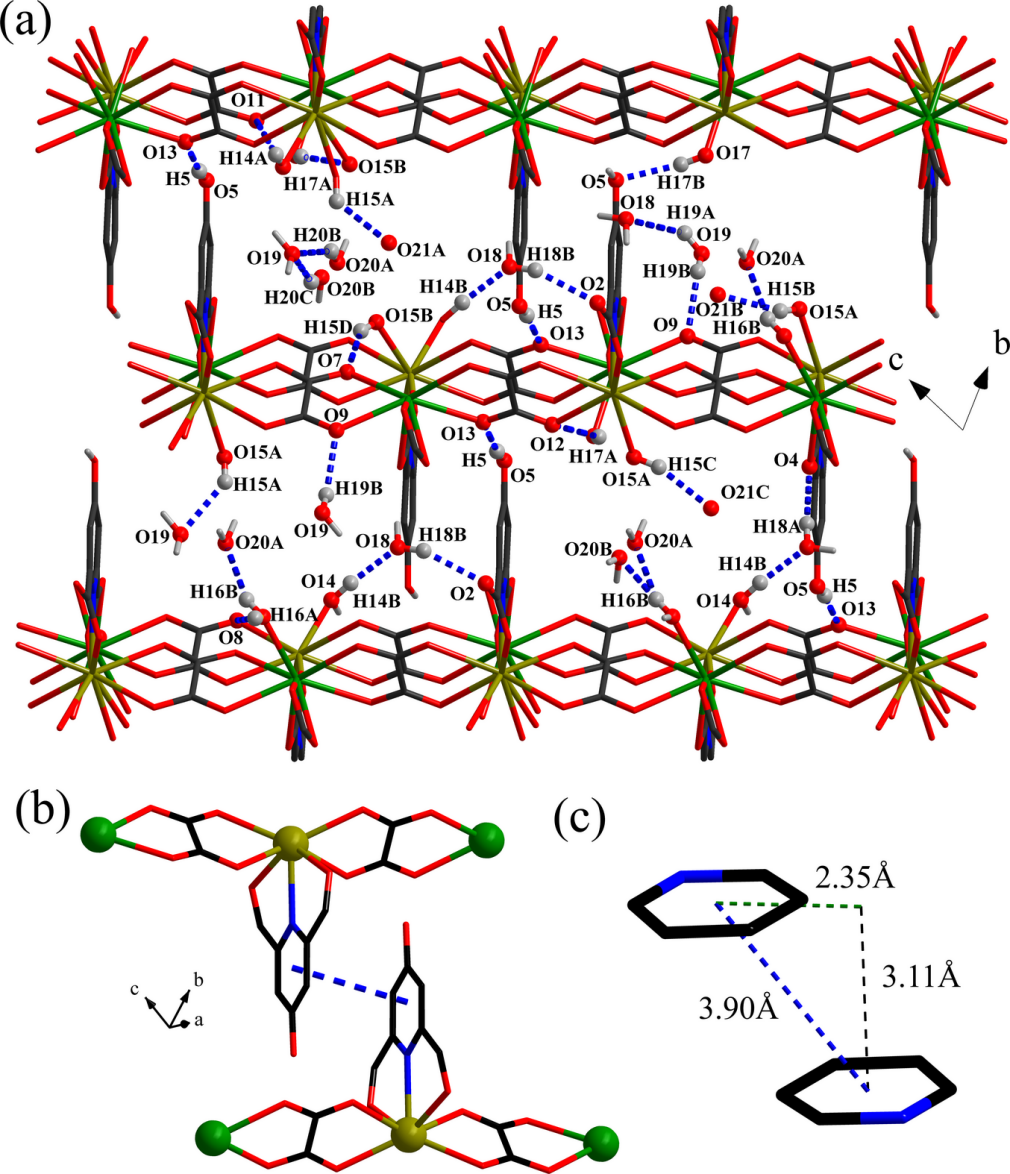
Views of (*a*) hydrogen-bonding inter­actions in the crystal structure of title compound (only hydrogen atoms involving the hydrogen bonding inter­actions are shown), (*b*) inter­layer π–π inter­action, and (*c*) displaced π–π stacking geometry.

The Pr1 and Pr2 ions show two different coordination environments. Pr1 has a nine-fold coordination environment defined by one pyridyl nitro­gen atom of HCAM^2−^ and eight oxygen atoms from one HCAM^2−^, two ox^2−^, and two coordinating water mol­ecules, leading to the formation of a tricapped trigonal–prismatic building unit, *i.e. TPRS*-{Pr^III^NO_8_} (Fig. 1 ▸*b*). Pr2 is tenfold coordinated to oxygen atoms from two HCAM^2−^, two ox^2−^, and two coordinating water mol­ecules, forming a bicapped square anti­prism, *i.e. SAPRS*-{Pr^III^O_10_} (Fig. 1 ▸*c*). The Pr—O bond lengths are in the range of 2.478 (3)–2.550 (3) Å (Table 1 ▸), which agrees well with those for other reported Pr^III^ frameworks containing HCAM^2−^ and ox^2−^ (Chen *et al.*, 2008 ▸; Zou *et al.*, 2009 ▸, 2010 ▸, 2011 ▸; Zhao *et al.*, 2009 ▸). The HCAM^2−^ linker has a μ_2_-κ^2^:κ^1^ chelating coordination mode *via* both carboxyl­ate groups and the N-pyridyl donor, thus creating infinite chains made up from alternating and edge-sharing *TPRS*-{Pr^III^NO_8_} and *SAPRS*-{Pr^III^O_10_} units that extend along [100]. The chains are connected to adjacent chains through bridging carboxyl­ates of the ox^2−^ linkers that adopt the common μ_2_-κ^1^:κ^1^:κ^1^:κ^1^ mode of coordination (Fig. 1 ▸*e*) along the *c*-axis, resulting in sheets in the *ac* plane. Neighboring sheets are connected to one another through both hydrogen-bonding inter­actions involving the coordinating and crystallizing water mol­ecules (Table 2 ▸, Fig. 2 ▸*a*) and inter­molecular π–π inter­actions between the pyridyl rings of the HCAM^2−^ ligands that protrude from the parallel sheets (Fig. 2 ▸*b*), leading to formation of a tri-periodic supra­molecular network. The π–π inter­actions are slightly offset from each other (Banerjee *et al.*, 2019 ▸; Yao *et al.*, 2018 ▸) and have a centroid-to-centroid distance of *ca* 3.90 Å, an offset distance of *ca* 2.35 Å, and are exactly parallel (Fig. 2 ▸*c*). The inter­planar stacking distance is 3.11 Å. The supra­molecular architecture established by these stabilizing inter­actions has an inter­layer distance between parallel sheets of *ca* 9.33 Å. Disregarding the co-crystallized not metal-coordinating water mol­ecules, the total potential solvent area volume within the inter­layer space is estimated to be *ca* 20% of the unit-cell volume, based on a calculation performed by *PLATON* software (Spek, 2020 ▸).

## Synthesis and crystallization

All chemicals used in this work were obtained commercially and used without purification: Pr_6_O_11_ (TJTM, 99.99%), chelidamic acid (H_3_CAM; C_7_H_5_NO_5_, Macklin, 98%), oxalic acid (H_2_ox·2H_2_O; C_2_H_2_O_4_·2H_2_O, Fluka Chemika, ≥99%), nitric acid (HNO_3_, RCI Labscan, 65%). Pr^III^(NO_3_)_3_·6H_2_O was prepared by dissolving Pr_6_O_11_ in small amount of concentrated solution of nitric acid followed by slow crystallization.

To synthesize the title compound, a mixture of Pr^III^(NO_3_)_3_·6H_2_O (43.5 mg, 0.100 mmol), H_3_CAM (20.5 mg, 0.100 mmol) and oxalic acid (12.6 mg, 0.100 mmol) was prepared in 10.0 ml of deionized water. The mixture was then transferred to a Teflon lined autoclave and heated at 130°C for 5 d under autogenous pressure. After cooling down to room temperature, brown block-shaped crystals (34% yield based on Pr^III^) were obtained, collected and washed with deionized water. The crystals were characterized using FT–IR spectroscopy (PerkinElmer/Frontier FT–IR instrument; ATR mode; cm^−1^): 3675–2814(*br*), 1647(*m*), 1562(*s*), 1443(*m*), 1396(*m*), 1354(*m*), 1305(*m*), 1251(*w*), 1121(*w*), 1027(*m*), 750(*m*), 486(*m*).

## Refinement

Crystal data, data collection and structure refinement details are summarized in Table 3 ▸.

The oxygen atom of a coordinating water mol­ecule (O15) shows site disorder splitting to two sites of O15*A* and O15*B* with site occupancies of 0.45 (4) and 0.55 (4), respectively. A SIMU command with an effective standard deviation of 0.01 Å^2^ was used to restrain O15*A* and O15*B* to have similar *U*^ij^ components. An oxygen atom of a crystallizing water mol­ecule (O20) is hydrogen bonded to its symmetry-equivalent counterpart which across a nearby inversion center (0.5 0 0.5), inducing disorder and splitting into two sites (O20*A* and O20*B*). As O20*A* and O20*B* are too close to be compatible with each other, one of them was moved across the inversion center. A SIMU (*s* = 0.01, *st* = 0.01, *d*_max_ = 3) together with a ISOR (*s* = 0.01, *st* = 0.02) command were applied to O20*A* and O20*B* to restrain their *U*^ij^ components to approximate isotropic behavior. The region where O21*A*, O21*B* and O21*C* of water mol­ecules of crystallization were placed originally contained several large electron densities, with O21*B* being in hydrogen-bonding distance to its own counterpart by inversion. The large number of permutations prevented an exact disorder modeling or placement of hydrogen atoms. However, the total number of site occupancies in this region was estimated to be about one and a third (1.294). A SIMU (*s* = 0.01, *st* = 0.02, *d*_max_ = 2) command was applied to O21A, O21B and O21C to restrain to have similar *U*^ij^ components.

The carbon-bound hydrogen atoms were positioned geometrically and refined isotropically using a riding model (AFIX 43). The C—H bond lengths in the pyridyl ring of HCAM^2−^ were constrained to 0.93 Å [*U_iso_*(H) = 1.2*U_iso_*(C)]. A hydrogen atom of an –OH group of HCAM^2−^ was positioned geometrically and refined isotropically with allowing a rotation with a tetra­hedral C—O—H angle (AFIX 147) to best fit the experimental electron density. The O—H bond length of this –OH group was set to be 0.82 Å [*U_iso_*(H) = 1.5*U_iso_*(O)]. The hydrogen atoms of water mol­ecules (both coordinating and crystallizing water) were refined isotropically, and the O—H bond lengths and H⋯H distances were restrained to 0.84 (2) Å and 1.36 (2) Å, respectively, [*U_iso_*(H) = 1.5*U_iso_*(O)]. There were some hydrogen atoms of water mol­ecules that were additionally restrained based on hydrogen-bonding considerations, *i.e.* H19*B*⋯O9 and H20*A*⋯O20*B* distances were restrained to 2.15 (2) Å, and the H15*A*⋯O21*A* distance was restrained to 2.25 (2) Å. The hydrogen atoms of O20*A* and O20*B* were initially refined in the same manner while a damping factor was applied. In the final refinement cycles, the damping factor was removed and the hydrogen atoms were constrained to ride on their carrying oxygen atoms (AFIX 3).

## Supplementary Material

Crystal structure: contains datablock(s) I. DOI: 10.1107/S2414314625004560/zl4083sup1.cif

Structure factors: contains datablock(s) I. DOI: 10.1107/S2414314625004560/zl4083Isup2.hkl

CCDC reference: 2448088

Additional supporting information:  crystallographic information; 3D view; checkCIF report

## Figures and Tables

**Table 1:** Selected bond lengths (Å)

Pr1—O1	2.517 (3)	Pr2—O1^i^	2.746 (3)
Pr1—O3	2.526 (3)	Pr2—O2^i^	2.580 (3)
Pr1—O6	2.503 (3)	Pr2—O3^ii^	2.772 (3)
Pr1—O8	2.535 (3)	Pr2—O4^ii^	2.608 (3)
Pr1—O10	2.532 (3)	Pr2—O7	2.478 (3)
Pr1—O12	2.520 (3)	Pr2—O9	2.538 (3)
Pr1—O14	2.510 (3)	Pr2—O11^iii^	2.519 (3)
Pr1—O15*A*	2.523 (16)	Pr2—O13^iii^	2.550 (3)
Pr1—O15*B*	2.501 (16)	Pr2—O16	2.496 (4)
Pr1—N1	2.555 (4)	Pr2—O17	2.487 (3)

Symmetry codes: (i) 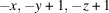; (ii) 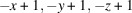; (iii) 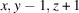.

**Table 2:** Hydrogen-bond geometry (Å, °)

*D*—H⋯*A*	*D*—H	H⋯*A*	*D*⋯*A*	*D*—H⋯*A*
O5—H5⋯O13^iv^	0.82	1.81	2.626 (4)	170
O14—H14*A*⋯O11^v^	0.82 (2)	1.92 (2)	2.735 (4)	173 (5)
O14—H14*B*⋯O18	0.83 (2)	1.93 (2)	2.755 (5)	175 (5)
O15*A*—H15*A*⋯O19^vi^	0.83 (2)	2.16 (4)	2.81 (3)	135 (3)
O15*A*—H15*A*⋯O21*A*	0.83 (2)	2.26 (2)	2.89 (2)	134 (4)
O15*A*—H15*B*⋯O21*B*	0.84 (2)	2.02 (9)	2.71 (3)	138 (11)
O15*B*—H15*C*⋯O21*C*	0.84 (2)	2.15 (6)	2.93 (3)	155 (10)
O15*B*—H15*D*⋯O7^ii^	0.86 (2)	1.96 (4)	2.75 (2)	152 (5)
O16—H16*A*⋯O8^i^	0.86 (2)	1.85 (2)	2.705 (4)	179 (5)
O16—H16*B*⋯O20*A*^vii^	0.83 (2)	2.17 (4)	2.96 (3)	159 (6)
O16—H16*B*⋯O20*B*	0.83 (2)	2.12 (3)	2.94 (2)	172 (5)
O17—H17*A*⋯O12^ii^	0.83 (2)	2.33 (4)	2.922 (4)	129 (4)
O17—H17*A*⋯O15*A*^ii^	0.83 (2)	2.13 (3)	2.892 (19)	153 (5)
O17—H17*A*⋯O15*B*^ii^	0.83 (2)	2.15 (3)	2.865 (18)	144 (4)
O17—H17*B*⋯O5^vii^	0.83 (2)	2.09 (2)	2.914 (4)	171 (5)
O18—H18*A*⋯O4^viii^	0.85 (2)	2.10 (2)	2.951 (6)	177 (6)
O18—H18*B*⋯O2^v^	0.85 (2)	2.21 (3)	3.011 (6)	156 (7)
O19—H19*A*⋯O18^vi^	0.83 (2)	2.56 (7)	3.161 (8)	130 (7)
O19—H19*B*⋯O9	0.83 (2)	2.15 (2)	2.904 (7)	151 (5)
O20*A*—H20*A*⋯O20*B*	0.87	2.17	2.901 (14)	141
O20*A*—H20*B*⋯O19^ix^	0.85	2.13	2.87 (3)	146
O20*B*—H20*C*⋯O19^i^	0.85	2.13	2.87 (2)	145

Symmetry codes: (i) 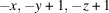; (ii) 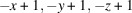; (iv) 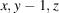; (v) 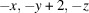; (vi) 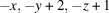; (vii) 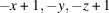; (viii) 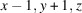; (ix) 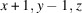.

**Table 3:** Experimental details

Crystal data	
Chemical formula	[Pr_2_(C_7_H_3_NO_5_)(C_2_O_4_)_2_(H_2_O)_4_]·4.29H_2_O
*M*_r_	785.78
Crystal system, space group	Triclinic, *P* 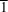
Temperature (K)	298
*a*, *b*, *c* (Å)	9.9236 (3), 10.3042 (4), 12.9748 (5)
α, β, γ (°)	66.684 (4), 80.942 (3), 66.660 (3)
*V* (Å^3^)	1118.69 (8)
*Z*	2
Radiation type	Mo *K*α
μ (mm^−1^)	4.41
Crystal size (mm)	0.2 × 0.2 × 0.1

Data collection	
Diffractometer	SuperNova, Single source at offset/far, HyPix3000
Absorption correction	Multi-scan (*CrysAlis PRO*; Rigaku OD, 2021 ▸)
*T*_min_, *T*_max_	0.768, 1.000
No. of measured, independent and observed [*I* > 2σ(*I*)] reflections	18710, 4715, 3799
*R*_int_	0.088
(sin θ/λ)_max_ (Å^−1^)	0.668

Refinement	
*R*[*F*^2^ > 2σ(*F*^2^)], *wR*(*F*^2^), *S*	0.036, 0.085, 1.05
No. of reflections	4715
No. of parameters	399
No. of restraints	71
H-atom treatment	H atoms treated by a mixture of independent and constrained refinement
Δρ_max_, Δρ_min_ (e Å^−3^)	1.47, −1.78

Computer programs: *CrysAlis PRO* (Rigaku OD, 2021 ▸), *SHELXT2018/2* (Sheldrick, 2015*a* ▸), *SHELXL2018/3* (Sheldrick, 2015*b* ▸) and *OLEX2* (Dolomanov *et al.*, 2009 ▸).

## full crystallographic data

### Poly[[tetraaqua(µ3-4-hydroxypyridine-2,6-dicarboxylato)di-µ2-oxalato-dipraseodymium(III)] 4.29-hydrate] Crystal data

**Table:**

[Pr_2_(C_7_H_3_NO_5_)(C_2_O_4_)_2_(H_2_O)_4_]·4.29H_2_O	*Z* = 2
*M**_r_* = 785.78	*F*(000) = 757
Triclinic, *P*1	*D*_x_ = 2.333 Mg m^−^^3^
*a* = 9.9236 (3) Å	Mo *K*α radiation, λ = 0.71073 Å
*b* = 10.3042 (4) Å	Cell parameters from 11399 reflections
*c* = 12.9748 (5) Å	θ = 2.3–27.2°
α = 66.684 (4)°	µ = 4.41 mm^−^^1^
β = 80.942 (3)°	*T* = 298 K
γ = 66.660 (3)°	Block, clear brownish colourless
*V* = 1118.69 (8) Å^3^	0.2 × 0.2 × 0.1 mm

### Poly[[tetraaqua(µ3-4-hydroxypyridine-2,6-dicarboxylato)di-µ2-oxalato-dipraseodymium(III)] 4.29-hydrate] Data collection

**Table:**

SuperNova, Single source at offset/far, HyPix3000 diffractometer	4715 independent reflections
Radiation source: micro-focus sealed X-ray tube, SuperNova (Mo) X-ray Source	3799 reflections with *I* > 2σ(*I*)
Detector resolution: 10.0000 pixels mm^-1^	*R*_int_ = 0.088
ω scans	θ_max_ = 28.3°, θ_min_ = 2.2°
Absorption correction: multi-scan (CrysAlisPro; Rigaku OD, 2021)	*h* = −12→12
*T*_min_ = 0.768, *T*_max_ = 1.000	*k* = −12→12
18710 measured reflections	*l* = −16→16

### Poly[[tetraaqua(µ3-4-hydroxypyridine-2,6-dicarboxylato)di-µ2-oxalato-dipraseodymium(III)] 4.29-hydrate] Refinement

**Table:**

Refinement on *F*^2^	Primary atom site location: dual
Least-squares matrix: full	Secondary atom site location: difference Fourier map
*R*[*F*^2^ > 2σ(*F*^2^)] = 0.036	Hydrogen site location: mixed
*wR*(*F*^2^) = 0.085	H atoms treated by a mixture of independent and constrained refinement
*S* = 1.05	*w* = 1/[σ^2^(*F*_o_^2^) + (0.0364*P*)^2^] where *P* = (*F*_o_^2^ + 2*F*_c_^2^)/3
4715 reflections	(Δ/σ)_max_ < 0.001
399 parameters	Δρ_max_ = 1.47 e Å^−^^3^
71 restraints	Δρ_min_ = −1.78 e Å^−^^3^

### Poly[[tetraaqua(µ3-4-hydroxypyridine-2,6-dicarboxylato)di-µ2-oxalato-dipraseodymium(III)] 4.29-hydrate] Special details

**Table:**

Geometry. All esds (except the esd in the dihedral angle between two l.s. planes) are estimated using the full covariance matrix. The cell esds are taken into account individually in the estimation of esds in distances, angles and torsion angles; correlations between esds in cell parameters are only used when they are defined by crystal symmetry. An approximate (isotropic) treatment of cell esds is used for estimating esds involving l.s. planes.

### Poly[[tetraaqua(µ3-4-hydroxypyridine-2,6-dicarboxylato)di-µ2-oxalato-dipraseodymium(III)] 4.29-hydrate] Fractional atomic coordinates and isotropic or equivalent isotropic displacement parameters (Å^2^)

**Table:**

	*x*	*y*	*z*	*U*_iso_*/*U*_eq_	Occ. (<1)
Pr1	0.23196 (2)	0.77685 (3)	0.25657 (2)	0.01644 (9)	
Pr2	0.23512 (2)	0.29264 (3)	0.76177 (2)	0.01623 (9)	
O1	0.0556 (3)	0.6667 (3)	0.2380 (3)	0.0213 (7)	
O2	−0.0132 (3)	0.5827 (4)	0.1307 (3)	0.0307 (8)	
O3	0.5023 (3)	0.6898 (3)	0.2099 (3)	0.0216 (7)	
O4	0.7077 (3)	0.5102 (4)	0.1918 (3)	0.0289 (8)	
O5	0.4995 (3)	0.2189 (4)	0.0567 (3)	0.0259 (8)	
H5	0.433892	0.212745	0.029681	0.039*	
O6	0.3619 (3)	0.5268 (4)	0.4061 (3)	0.0304 (8)	
O7	0.3719 (3)	0.3733 (4)	0.5863 (3)	0.0252 (8)	
O8	0.0950 (3)	0.7142 (3)	0.4408 (3)	0.0256 (8)	
O9	0.0969 (3)	0.5504 (4)	0.6168 (3)	0.0275 (8)	
O10	0.1736 (3)	0.8820 (3)	0.0496 (2)	0.0213 (7)	
O11	0.1805 (3)	1.0576 (3)	−0.1203 (2)	0.0216 (7)	
O12	0.2910 (3)	1.0092 (3)	0.1385 (3)	0.0242 (7)	
O13	0.3127 (3)	1.1706 (3)	−0.0329 (2)	0.0234 (7)	
O14	−0.0079 (4)	0.9973 (4)	0.2305 (3)	0.0302 (8)	
H14A	−0.066 (5)	0.983 (6)	0.201 (4)	0.045*	
H14B	−0.016 (5)	1.088 (3)	0.200 (4)	0.045*	
O15A	0.298 (3)	0.894 (3)	0.3700 (17)	0.028 (4)	0.45 (4)
H15A	0.234 (5)	0.956 (5)	0.395 (3)	0.043*	0.45 (4)
H15B	0.369 (8)	0.842 (8)	0.415 (8)	0.043*	0.45 (4)
O15B	0.342 (2)	0.828 (3)	0.3904 (16)	0.042 (4)	0.55 (4)
H15C	0.293 (5)	0.859 (12)	0.440 (6)	0.064*	0.55 (4)
H15D	0.427 (5)	0.767 (8)	0.420 (6)	0.064*	0.55 (4)
O16	0.1824 (4)	0.2134 (5)	0.6187 (3)	0.0400 (10)	
H16A	0.094 (3)	0.236 (6)	0.601 (4)	0.060*	
H16B	0.233 (5)	0.209 (7)	0.562 (3)	0.060*	
O17	0.4416 (3)	0.0634 (4)	0.7485 (3)	0.0304 (9)	
H17A	0.522 (3)	0.073 (5)	0.736 (4)	0.046*	
H17B	0.454 (5)	−0.020 (3)	0.800 (3)	0.046*	
O18	−0.0349 (5)	1.2962 (5)	0.1190 (4)	0.0638 (13)	
H18A	−0.109 (5)	1.355 (6)	0.143 (5)	0.096*	
H18B	−0.017 (7)	1.350 (6)	0.053 (3)	0.096*	
O19	−0.1396 (7)	0.7992 (7)	0.6626 (6)	0.106 (2)	
H19A	−0.150 (8)	0.811 (12)	0.724 (5)	0.159*	
H19B	−0.055 (4)	0.743 (7)	0.655 (8)	0.159*	
O20A	0.599 (3)	−0.1133 (16)	0.544 (2)	0.126 (6)	0.5
H20A	0.516875	−0.037798	0.542562	0.189*	0.5
H20B	0.661995	−0.098201	0.570062	0.189*	0.5
O20B	0.389 (3)	0.1898 (16)	0.4318 (19)	0.112 (5)	0.5
H20C	0.299245	0.227002	0.411383	0.168*	0.5
H20D	0.402974	0.255335	0.447541	0.168*	0.5
O21A	0.1227 (19)	0.9358 (19)	0.5629 (13)	0.131 (6)	0.488 (17)
O21C	0.214 (3)	0.838 (3)	0.608 (2)	0.108 (6)	0.253 (13)
O21B	0.3984 (11)	0.7322 (11)	0.5831 (8)	0.097 (5)	0.553 (15)
N1	0.3350 (4)	0.5635 (4)	0.1792 (3)	0.0188 (8)	
C1	0.0848 (5)	0.5939 (5)	0.1735 (4)	0.0228 (11)	
C2	0.2449 (4)	0.5154 (5)	0.1506 (4)	0.0171 (10)	
C3	0.2935 (5)	0.4015 (5)	0.1089 (4)	0.0218 (10)	
H3	0.226867	0.373002	0.088373	0.026*	
C4	0.4425 (5)	0.3294 (5)	0.0978 (4)	0.0194 (10)	
C5	0.5398 (5)	0.3766 (5)	0.1287 (4)	0.0237 (11)	
H5A	0.640885	0.328970	0.123911	0.028*	
C6	0.4805 (4)	0.4958 (5)	0.1664 (4)	0.0181 (10)	
C7	0.5711 (5)	0.5680 (5)	0.1928 (4)	0.0193 (10)	
C9	0.1529 (5)	0.5941 (5)	0.5226 (4)	0.0193 (10)	
C8	0.3103 (5)	0.4884 (5)	0.5036 (4)	0.0202 (10)	
C11	0.2745 (5)	1.0625 (5)	0.0363 (4)	0.0195 (10)	
C10	0.2019 (5)	0.9932 (5)	−0.0159 (4)	0.0177 (10)	

### Poly[[tetraaqua(µ3-4-hydroxypyridine-2,6-dicarboxylato)di-µ2-oxalato-dipraseodymium(III)] 4.29-hydrate] Atomic displacement parameters (Å^2^)

**Table:**

	*U*^11^	*U*^22^	*U*^33^	*U*^12^	*U*^13^	*U*^23^
Pr1	0.01715 (16)	0.01723 (16)	0.01250 (17)	−0.00734 (12)	0.00045 (11)	−0.00222 (12)
Pr2	0.01536 (16)	0.01812 (15)	0.01234 (16)	−0.00743 (12)	0.00053 (11)	−0.00168 (12)
O1	0.0189 (16)	0.0243 (18)	0.0231 (19)	−0.0089 (14)	0.0049 (13)	−0.0122 (15)
O2	0.0163 (17)	0.043 (2)	0.045 (2)	−0.0117 (16)	0.0039 (15)	−0.0283 (18)
O3	0.0233 (18)	0.0244 (18)	0.0215 (19)	−0.0128 (15)	0.0024 (14)	−0.0097 (15)
O4	0.0166 (18)	0.032 (2)	0.042 (2)	−0.0098 (15)	0.0002 (15)	−0.0170 (18)
O5	0.0178 (17)	0.0267 (18)	0.038 (2)	−0.0046 (15)	−0.0057 (15)	−0.0177 (16)
O6	0.0258 (19)	0.034 (2)	0.0159 (19)	−0.0058 (16)	0.0034 (14)	−0.0006 (16)
O7	0.0206 (17)	0.0287 (19)	0.0137 (18)	−0.0078 (15)	−0.0005 (13)	0.0034 (15)
O8	0.0241 (18)	0.0224 (18)	0.0198 (19)	−0.0068 (15)	0.0003 (14)	0.0008 (15)
O9	0.0251 (18)	0.033 (2)	0.0153 (19)	−0.0090 (15)	0.0067 (14)	−0.0035 (15)
O10	0.0301 (18)	0.0207 (17)	0.0138 (18)	−0.0158 (15)	−0.0012 (13)	−0.0001 (14)
O11	0.0257 (18)	0.0258 (18)	0.0132 (18)	−0.0136 (15)	−0.0039 (13)	−0.0016 (14)
O12	0.0335 (19)	0.0266 (18)	0.0133 (19)	−0.0168 (15)	−0.0052 (14)	−0.0004 (15)
O13	0.0284 (18)	0.0259 (18)	0.0181 (19)	−0.0177 (15)	−0.0024 (14)	−0.0017 (15)
O14	0.027 (2)	0.0250 (19)	0.040 (2)	−0.0093 (16)	−0.0066 (16)	−0.0112 (18)
O15A	0.029 (9)	0.042 (9)	0.023 (7)	−0.012 (7)	0.004 (5)	−0.023 (7)
O15B	0.028 (7)	0.060 (11)	0.046 (7)	−0.016 (7)	0.000 (5)	−0.027 (8)
O16	0.026 (2)	0.069 (3)	0.039 (3)	−0.019 (2)	0.0060 (18)	−0.034 (2)
O17	0.0188 (18)	0.0220 (19)	0.041 (2)	−0.0083 (15)	0.0046 (16)	−0.0036 (16)
O18	0.075 (4)	0.036 (3)	0.070 (4)	−0.012 (2)	0.003 (3)	−0.019 (2)
O19	0.098 (4)	0.087 (4)	0.116 (5)	−0.008 (4)	0.010 (4)	−0.049 (4)
O20A	0.125 (7)	0.110 (13)	0.150 (10)	−0.017 (11)	−0.032 (7)	−0.069 (11)
O20B	0.124 (7)	0.097 (12)	0.131 (10)	−0.017 (10)	−0.027 (7)	−0.071 (11)
O21A	0.160 (12)	0.094 (10)	0.119 (10)	−0.013 (8)	−0.012 (8)	−0.048 (8)
O21C	0.145 (11)	0.082 (9)	0.089 (9)	−0.011 (8)	−0.020 (8)	−0.047 (8)
O21B	0.115 (8)	0.081 (7)	0.063 (7)	−0.006 (6)	−0.017 (5)	−0.019 (5)
N1	0.015 (2)	0.021 (2)	0.019 (2)	−0.0091 (17)	0.0012 (16)	−0.0049 (17)
C1	0.019 (3)	0.018 (2)	0.029 (3)	−0.009 (2)	0.001 (2)	−0.004 (2)
C2	0.014 (2)	0.023 (2)	0.014 (2)	−0.0065 (19)	−0.0006 (18)	−0.007 (2)
C3	0.023 (3)	0.028 (3)	0.022 (3)	−0.014 (2)	−0.001 (2)	−0.011 (2)
C4	0.024 (3)	0.014 (2)	0.017 (3)	−0.007 (2)	−0.0011 (19)	−0.002 (2)
C5	0.015 (2)	0.026 (3)	0.027 (3)	−0.005 (2)	−0.005 (2)	−0.007 (2)
C6	0.018 (2)	0.017 (2)	0.018 (3)	−0.0061 (19)	−0.0011 (19)	−0.005 (2)
C7	0.018 (3)	0.023 (3)	0.016 (3)	−0.011 (2)	−0.0003 (19)	−0.002 (2)
C9	0.016 (2)	0.023 (3)	0.017 (3)	−0.006 (2)	0.0000 (19)	−0.007 (2)
C8	0.016 (2)	0.024 (3)	0.020 (3)	−0.008 (2)	0.001 (2)	−0.007 (2)
C11	0.015 (2)	0.023 (3)	0.019 (3)	−0.008 (2)	−0.0031 (19)	−0.004 (2)
C10	0.015 (2)	0.020 (2)	0.019 (3)	−0.0051 (19)	−0.0039 (18)	−0.007 (2)

### Poly[[tetraaqua(µ3-4-hydroxypyridine-2,6-dicarboxylato)di-µ2-oxalato-dipraseodymium(III)] 4.29-hydrate] Geometric parameters (Å, º)

**Table:**

Pr1—O1	2.517 (3)	O11—C10	1.260 (5)
Pr1—O3	2.526 (3)	O12—C11	1.227 (5)
Pr1—O6	2.503 (3)	O13—C11	1.275 (5)
Pr1—O8	2.535 (3)	O14—H14A	0.821 (19)
Pr1—O10	2.532 (3)	O14—H14B	0.832 (19)
Pr1—O12	2.520 (3)	O15A—H15A	0.830 (19)
Pr1—O14	2.510 (3)	O15A—H15B	0.84 (2)
Pr1—O15A	2.523 (16)	O15B—H15C	0.84 (2)
Pr1—O15B	2.501 (16)	O15B—H15D	0.86 (2)
Pr1—N1	2.555 (4)	O16—H16A	0.856 (19)
Pr2—O1^i^	2.746 (3)	O16—H16B	0.826 (19)
Pr2—O2^i^	2.580 (3)	O17—H17A	0.826 (18)
Pr2—O3^ii^	2.772 (3)	O17—H17B	0.832 (19)
Pr2—O4^ii^	2.608 (3)	O18—H18A	0.85 (2)
Pr2—O7	2.478 (3)	O18—H18B	0.85 (2)
Pr2—O9	2.538 (3)	O19—H19A	0.83 (2)
Pr2—O11^iii^	2.519 (3)	O19—H19B	0.831 (19)
Pr2—O13^iii^	2.550 (3)	O20A—H20A	0.8688
Pr2—O16	2.496 (4)	O20A—H20B	0.8471
Pr2—O17	2.487 (3)	O20B—H20C	0.8540
Pr2—C1^i^	3.025 (4)	O20B—H20D	0.8435
Pr2—C7^ii^	3.054 (4)	N1—C2	1.336 (5)
O1—C1	1.263 (5)	N1—C6	1.348 (5)
O2—C1	1.256 (5)	C1—C2	1.513 (6)
O3—C7	1.259 (5)	C2—C3	1.370 (6)
O4—C7	1.247 (5)	C3—H3	0.9300
O5—H5	0.8200	C3—C4	1.381 (6)
O5—C4	1.333 (5)	C4—C5	1.407 (6)
O6—C8	1.256 (5)	C5—H5A	0.9300
O7—C8	1.248 (5)	C5—C6	1.381 (6)
O8—C9	1.256 (5)	C6—C7	1.519 (6)
O9—C9	1.247 (5)	C9—C8	1.559 (7)
O10—C10	1.235 (5)	C11—C10	1.559 (6)
			
O1—Pr1—O3	125.52 (9)	O16—Pr2—O9	79.58 (12)
O1—Pr1—O8	71.98 (10)	O16—Pr2—O11^iii^	77.00 (11)
O1—Pr1—O10	73.53 (9)	O16—Pr2—O13^iii^	137.51 (12)
O1—Pr1—O12	134.40 (10)	O16—Pr2—C1^i^	92.58 (12)
O1—Pr1—O15A	144.9 (4)	O16—Pr2—C7^ii^	142.54 (11)
O1—Pr1—N1	63.08 (10)	O17—Pr2—O1^i^	123.96 (10)
O3—Pr1—O8	131.86 (10)	O17—Pr2—O2^i^	150.18 (10)
O3—Pr1—O10	89.35 (9)	O17—Pr2—O3^ii^	70.95 (10)
O3—Pr1—N1	62.46 (10)	O17—Pr2—O4^ii^	119.01 (10)
O6—Pr1—O1	88.21 (10)	O17—Pr2—O9	133.08 (10)
O6—Pr1—O3	71.33 (10)	O17—Pr2—O11^iii^	70.13 (10)
O6—Pr1—O8	64.40 (10)	O17—Pr2—O13^iii^	82.94 (11)
O6—Pr1—O10	138.63 (11)	O17—Pr2—O16	69.13 (12)
O6—Pr1—O12	135.43 (9)	O17—Pr2—C1^i^	142.91 (10)
O6—Pr1—O14	138.35 (11)	O17—Pr2—C7^ii^	95.28 (11)
O6—Pr1—O15A	85.8 (7)	C1^i^—Pr2—C7^ii^	116.61 (12)
O6—Pr1—N1	68.80 (11)	Pr1—O1—Pr2^i^	144.80 (13)
O8—Pr1—N1	114.36 (10)	C1—O1—Pr1	120.3 (3)
O10—Pr1—O8	137.05 (9)	C1—O1—Pr2^i^	90.1 (2)
O10—Pr1—N1	69.83 (10)	C1—O2—Pr2^i^	98.1 (3)
O12—Pr1—O3	72.10 (10)	Pr1—O3—Pr2^ii^	143.18 (13)
O12—Pr1—O8	131.56 (10)	C7—O3—Pr1	123.1 (3)
O12—Pr1—O10	64.34 (9)	C7—O3—Pr2^ii^	90.4 (2)
O12—Pr1—O15A	66.4 (5)	C7—O4—Pr2^ii^	98.6 (3)
O12—Pr1—N1	114.05 (10)	C4—O5—H5	109.5
O14—Pr1—O1	78.28 (10)	C8—O6—Pr1	121.4 (3)
O14—Pr1—O3	146.68 (10)	C8—O7—Pr2	121.3 (3)
O14—Pr1—O8	73.95 (10)	C9—O8—Pr1	120.6 (3)
O14—Pr1—O10	74.65 (11)	C9—O9—Pr2	119.4 (3)
O14—Pr1—O12	74.62 (10)	C10—O10—Pr1	120.7 (3)
O14—Pr1—O15A	83.6 (6)	C10—O11—Pr2^iv^	123.2 (3)
O14—Pr1—N1	133.09 (11)	C11—O12—Pr1	120.1 (3)
O15A—Pr1—O3	84.8 (5)	C11—O13—Pr2^iv^	121.0 (3)
O15A—Pr1—O8	74.3 (4)	Pr1—O14—H14A	108 (4)
O15A—Pr1—O10	129.7 (6)	Pr1—O14—H14B	124 (3)
O15A—Pr1—N1	143.2 (6)	H14A—O14—H14B	110 (5)
O15B—Pr1—O1	144.6 (4)	Pr1—O15A—H15A	122 (3)
O15B—Pr1—O3	76.7 (5)	Pr1—O15A—H15B	121 (3)
O15B—Pr1—O6	72.4 (7)	H15A—O15A—H15B	109 (3)
O15B—Pr1—O8	72.9 (4)	Pr1—O15B—H15C	123 (3)
O15B—Pr1—O10	139.4 (5)	Pr1—O15B—H15D	121 (3)
O15B—Pr1—O12	75.1 (6)	H15C—O15B—H15D	105 (3)
O15B—Pr1—O14	96.5 (6)	Pr2—O16—H16A	121 (3)
O15B—Pr1—N1	130.4 (6)	Pr2—O16—H16B	124 (3)
O1^i^—Pr2—O3^ii^	164.06 (9)	H16A—O16—H16B	107 (6)
O1^i^—Pr2—C1^i^	24.67 (10)	Pr2—O17—H17A	114 (4)
O1^i^—Pr2—C7^ii^	140.12 (11)	Pr2—O17—H17B	119 (3)
O2^i^—Pr2—O1^i^	48.86 (9)	H17A—O17—H17B	107 (3)
O2^i^—Pr2—O3^ii^	115.62 (9)	H18A—O18—H18B	106 (3)
O2^i^—Pr2—O4^ii^	72.16 (9)	H19A—O19—H19B	112 (4)
O2^i^—Pr2—C1^i^	24.28 (11)	H20A—O20A—H20B	105.4
O2^i^—Pr2—C7^ii^	93.86 (11)	H20C—O20B—H20D	107.1
O3^ii^—Pr2—C1^i^	139.57 (11)	C2—N1—Pr1	120.4 (3)
O3^ii^—Pr2—C7^ii^	24.35 (10)	C2—N1—C6	118.0 (4)
O4^ii^—Pr2—O1^i^	116.38 (9)	C6—N1—Pr1	121.6 (3)
O4^ii^—Pr2—O3^ii^	48.16 (9)	O1—C1—Pr2^i^	65.2 (2)
O4^ii^—Pr2—C1^i^	93.52 (11)	O1—C1—C2	117.6 (4)
O4^ii^—Pr2—C7^ii^	23.81 (11)	O2—C1—Pr2^i^	57.6 (2)
O7—Pr2—O1^i^	122.36 (9)	O2—C1—O1	122.4 (4)
O7—Pr2—O2^i^	137.91 (11)	O2—C1—C2	120.0 (4)
O7—Pr2—O3^ii^	64.97 (9)	C2—C1—Pr2^i^	172.1 (3)
O7—Pr2—O4^ii^	84.16 (10)	N1—C2—C1	113.6 (4)
O7—Pr2—O9	64.76 (10)	N1—C2—C3	123.1 (4)
O7—Pr2—O11^iii^	136.21 (10)	C3—C2—C1	123.2 (4)
O7—Pr2—O13^iii^	131.51 (9)	C2—C3—H3	120.4
O7—Pr2—O16	69.72 (11)	C2—C3—C4	119.2 (4)
O7—Pr2—O17	71.87 (10)	C4—C3—H3	120.4
O7—Pr2—C1^i^	133.16 (11)	O5—C4—C3	123.3 (4)
O7—Pr2—C7^ii^	73.09 (11)	O5—C4—C5	117.9 (4)
O9—Pr2—O1^i^	70.34 (9)	C3—C4—C5	118.8 (4)
O9—Pr2—O2^i^	75.37 (10)	C4—C5—H5A	121.1
O9—Pr2—O3^ii^	104.33 (9)	C6—C5—C4	117.9 (4)
O9—Pr2—O4^ii^	74.22 (10)	C6—C5—H5A	121.1
O9—Pr2—O13^iii^	140.30 (10)	N1—C6—C5	123.0 (4)
O9—Pr2—C1^i^	69.61 (11)	N1—C6—C7	113.3 (4)
O9—Pr2—C7^ii^	88.79 (11)	C5—C6—C7	123.7 (4)
O11^iii^—Pr2—O1^i^	66.23 (9)	O3—C7—Pr2^ii^	65.2 (2)
O11^iii^—Pr2—O2^i^	82.52 (10)	O3—C7—C6	117.1 (4)
O11^iii^—Pr2—O3^ii^	119.82 (9)	O4—C7—Pr2^ii^	57.6 (2)
O11^iii^—Pr2—O4^ii^	133.81 (10)	O4—C7—O3	122.8 (4)
O11^iii^—Pr2—O9	135.73 (9)	O4—C7—C6	120.0 (4)
O11^iii^—Pr2—O13^iii^	63.37 (9)	C6—C7—Pr2^ii^	177.2 (3)
O11^iii^—Pr2—C1^i^	74.49 (10)	O8—C9—C8	116.4 (4)
O11^iii^—Pr2—C7^ii^	131.07 (11)	O9—C9—O8	126.7 (4)
O13^iii^—Pr2—O1^i^	106.12 (9)	O9—C9—C8	116.9 (4)
O13^iii^—Pr2—O2^i^	74.10 (10)	O6—C8—C9	116.7 (4)
O13^iii^—Pr2—O3^ii^	67.88 (9)	O7—C8—O6	126.5 (4)
O13^iii^—Pr2—O4^ii^	72.69 (10)	O7—C8—C9	116.8 (4)
O13^iii^—Pr2—C1^i^	91.14 (12)	O12—C11—O13	125.7 (4)
O13^iii^—Pr2—C7^ii^	68.71 (10)	O12—C11—C10	118.3 (4)
O16—Pr2—O1^i^	68.24 (10)	O13—C11—C10	115.9 (4)
O16—Pr2—O2^i^	116.84 (10)	O10—C10—O11	128.1 (4)
O16—Pr2—O3^ii^	126.51 (10)	O10—C10—C11	116.3 (4)
O16—Pr2—O4^ii^	149.02 (12)	O11—C10—C11	115.6 (4)
			
Pr1—O1—C1—Pr2^i^	−161.4 (3)	Pr2^iv^—O11—C10—C11	−3.7 (5)
Pr1—O1—C1—O2	−154.6 (3)	Pr2^iv^—O13—C11—O12	−170.6 (4)
Pr1—O1—C1—C2	26.9 (5)	Pr2^iv^—O13—C11—C10	9.8 (5)
Pr1—O3—C7—Pr2^ii^	−163.8 (3)	O1—C1—C2—N1	−15.8 (6)
Pr1—O3—C7—O4	−165.4 (3)	O1—C1—C2—C3	161.7 (4)
Pr1—O3—C7—C6	17.9 (5)	O2—C1—C2—N1	165.7 (4)
Pr1—O6—C8—O7	−173.3 (3)	O2—C1—C2—C3	−16.9 (6)
Pr1—O6—C8—C9	7.5 (5)	O5—C4—C5—C6	177.0 (4)
Pr1—O8—C9—O9	−179.6 (3)	O8—C9—C8—O6	−4.7 (6)
Pr1—O8—C9—C8	−0.3 (5)	O8—C9—C8—O7	176.0 (4)
Pr1—O10—C10—O11	179.5 (3)	O9—C9—C8—O6	174.6 (4)
Pr1—O10—C10—C11	0.1 (5)	O9—C9—C8—O7	−4.6 (6)
Pr1—O12—C11—O13	−173.4 (3)	O12—C11—C10—O10	−4.2 (7)
Pr1—O12—C11—C10	6.1 (6)	O12—C11—C10—O11	176.3 (4)
Pr1—N1—C2—C1	−2.4 (5)	O13—C11—C10—O10	175.3 (4)
Pr1—N1—C2—C3	−179.9 (3)	O13—C11—C10—O11	−4.1 (6)
Pr1—N1—C6—C5	177.6 (3)	N1—C2—C3—C4	1.5 (7)
Pr1—N1—C6—C7	−4.8 (5)	N1—C6—C7—O3	−8.1 (6)
Pr2^i^—O1—C1—O2	6.8 (4)	N1—C6—C7—O4	175.1 (4)
Pr2^i^—O1—C1—C2	−171.7 (3)	C1—C2—C3—C4	−175.7 (4)
Pr2^i^—O2—C1—O1	−7.3 (5)	C2—N1—C6—C5	−2.1 (6)
Pr2^i^—O2—C1—C2	171.2 (3)	C2—N1—C6—C7	175.5 (4)
Pr2^ii^—O3—C7—O4	−1.6 (4)	C2—C3—C4—O5	−179.1 (4)
Pr2^ii^—O3—C7—C6	−178.3 (3)	C2—C3—C4—C5	−0.6 (6)
Pr2^ii^—O4—C7—O3	1.7 (5)	C3—C4—C5—C6	−1.5 (6)
Pr2^ii^—O4—C7—C6	178.3 (3)	C4—C5—C6—N1	3.0 (7)
Pr2—O7—C8—O6	−168.6 (3)	C4—C5—C6—C7	−174.4 (4)
Pr2—O7—C8—C9	10.6 (5)	C5—C6—C7—O3	169.5 (4)
Pr2—O9—C9—O8	175.9 (3)	C5—C6—C7—O4	−7.3 (7)
Pr2—O9—C9—C8	−3.3 (5)	C6—N1—C2—C1	177.3 (4)
Pr2^iv^—O11—C10—O10	176.9 (3)	C6—N1—C2—C3	−0.2 (6)

Symmetry codes: (i) −*x*, −*y*+1, −*z*+1; (ii) −*x*+1, −*y*+1, −*z*+1; (iii) *x*, *y*−1, *z*+1; (iv) *x*, *y*+1, *z*−1.

### Poly[[tetraaqua(µ3-4-hydroxypyridine-2,6-dicarboxylato)di-µ2-oxalato-dipraseodymium(III)] 4.29-hydrate] Hydrogen-bond geometry (Å, º)

**Table:**

*D*—H···*A*	*D*—H	H···*A*	*D*···*A*	*D*—H···*A*
O5—H5···O13^v^	0.82	1.81	2.626 (4)	170
O14—H14*A*···O11^vi^	0.82 (2)	1.92 (2)	2.735 (4)	173 (5)
O14—H14*B*···O18	0.83 (2)	1.93 (2)	2.755 (5)	175 (5)
O15*A*—H15*A*···O19^vii^	0.83 (2)	2.16 (4)	2.81 (3)	135 (3)
O15*A*—H15*A*···O21*A*	0.83 (2)	2.26 (2)	2.89 (2)	134 (4)
O15*A*—H15*B*···O21*B*	0.84 (2)	2.02 (9)	2.71 (3)	138 (11)
O15*B*—H15*C*···O21*C*	0.84 (2)	2.15 (6)	2.93 (3)	155 (10)
O15*B*—H15*D*···O7^ii^	0.86 (2)	1.96 (4)	2.75 (2)	152 (5)
O16—H16*A*···O8^i^	0.86 (2)	1.85 (2)	2.705 (4)	179 (5)
O16—H16*B*···O20*A*^viii^	0.83 (2)	2.17 (4)	2.96 (3)	159 (6)
O16—H16*B*···O20*B*	0.83 (2)	2.12 (3)	2.94 (2)	172 (5)
O17—H17*A*···O12^ii^	0.83 (2)	2.33 (4)	2.922 (4)	129 (4)
O17—H17*A*···O15*A*^ii^	0.83 (2)	2.13 (3)	2.892 (19)	153 (5)
O17—H17*A*···O15*B*^ii^	0.83 (2)	2.15 (3)	2.865 (18)	144 (4)
O17—H17*B*···O5^viii^	0.83 (2)	2.09 (2)	2.914 (4)	171 (5)
O18—H18*A*···O4^ix^	0.85 (2)	2.10 (2)	2.951 (6)	177 (6)
O18—H18*B*···O2^vi^	0.85 (2)	2.21 (3)	3.011 (6)	156 (7)
O19—H19*A*···O18^vii^	0.83 (2)	2.56 (7)	3.161 (8)	130 (7)
O19—H19*B*···O9	0.83 (2)	2.15 (2)	2.904 (7)	151 (5)
O20*A*—H20*A*···O20*B*	0.87	2.17	2.901 (14)	141
O20*A*—H20*B*···O19^x^	0.85	2.13	2.87 (3)	146
O20*B*—H20*C*···O19^i^	0.85	2.13	2.87 (2)	145

Symmetry codes: (i) −*x*, −*y*+1, −*z*+1; (ii) −*x*+1, −*y*+1, −*z*+1; (v) *x*, *y*−1, *z*; (vi) −*x*, −*y*+2, −*z*; (vii) −*x*, −*y*+2, −*z*+1; (viii) −*x*+1, −*y*, −*z*+1; (ix) *x*−1, *y*+1, *z*; (x) *x*+1, *y*−1, *z*.
